# *N*′-[(5-Methyl­furan-2-yl)methyl­ene]isonicotinohydrazide

**DOI:** 10.1107/S1600536808037276

**Published:** 2008-11-20

**Authors:** Li Liu, Fang-Fang Jian

**Affiliations:** a Microscale Science Institute, Weifang University, Weifang 261061, People’s Republic of China

## Abstract

The title compound, C_12_H_11_N_3_O_2_, was prepared by the reaction of isonicotinohydrazide and 5-methyl­furan-2-carbalde­hyde. The pyridine ring makes a dihedral angle of 46.90 (9)° with the mean plane of the furan ring. The crystal packing is stabilized by a bifurcated inter­molecular N—H⋯(N,O) inter­action.

## Related literature

For general background, see: Cimerman *et al.* (1997 ▶). For bond-length data, see: Chiu *et al.* (1998 ▶).
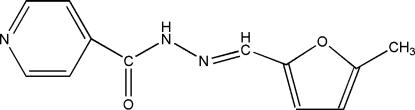


## Experimental

### 

#### Crystal data

C_12_H_11_N_3_O_2_
*M* *_r_* = 229.24
Tetragonal, 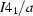

*a* = 17.313 (3) Å
*c* = 15.749 (5) Å
*V* = 4720.5 (18) Å^3^
*Z* = 16
Mo *K*α radiation
μ = 0.09 mm^−1^
*T* = 293 (2) K
0.25 × 0.20 × 0.19 mm

#### Data collection

Bruker SMART CCD area-detector diffractometer
Absorption correction: none
14901 measured reflections
2911 independent reflections
2151 reflections with *I* > 2σ(*I*)
*R*_int_ = 0.028

#### Refinement

*R*[*F* ^2^ > 2σ(*F* ^2^)] = 0.041
*wR*(*F* ^2^) = 0.117
*S* = 1.04
2911 reflections
155 parameters
H-atom parameters constrained
Δρ_max_ = 0.17 e Å^−3^
Δρ_min_ = −0.14 e Å^−3^

### 

Data collection: *SMART* (Bruker, 1997 ▶); cell refinement: *SAINT* (Bruker, 1997 ▶); data reduction: *SAINT*; program(s) used to solve structure: *SHELXS97* (Sheldrick, 2008 ▶); program(s) used to refine structure: *SHELXL97* (Sheldrick, 2008 ▶); molecular graphics: *SHELXTL* (Sheldrick, 2008 ▶); software used to prepare material for publication: *SHELXTL*.

## Supplementary Material

Crystal structure: contains datablocks global, I. DOI: 10.1107/S1600536808037276/at2662sup1.cif

Structure factors: contains datablocks I. DOI: 10.1107/S1600536808037276/at2662Isup2.hkl

Additional supplementary materials:  crystallographic information; 3D view; checkCIF report

## Figures and Tables

**Table 1:** Hydrogen-bond geometry (Å, °)

*D*—H⋯*A*	*D*—H	H⋯*A*	*D*⋯*A*	*D*—H⋯*A*
N2—H2*A*⋯O1^i^	0.86	2.18	2.9083 (19)	143
N2—H2*A*⋯N3^i^	0.86	2.58	3.3255 (19)	146

Symmetry code: (i) 
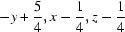
.

**Fig. 1.**
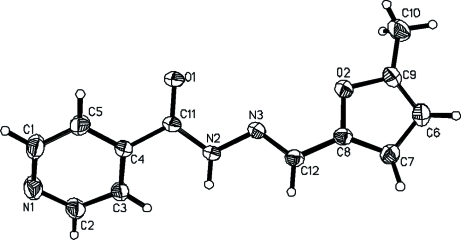
The molecular structure of (I) showing 30% probability displacement ellipsoids and the atom-numbering scheme.

## supplementary crystallographic information

### Comment

Schiff bases have received considerable attention in the literature. They are attractive from several points of view, such as the possibility of analytical application (Cimerman *et al.*, 1997). As part of our search for new schiff base compounds we synthesized the title compound (I), and describe its structure here.

In the title compound (I) (Fig. 1), the C12—N3 bond length of 1.2812 (17)Å is comparable with C—N double bond [1.284 (2) Å] reported (Chiu *et al.*, 1998). The pyridine ring (N1/C1–C5) makes a dihedral angle of 46.90 (9)°, with the plane of the furan ring (O2/C6–C9).

The crsytal packing is stabilized by intermolecular N—H···O, N—H···N hydrogen bonds (Table 1, Fig. 2).

### Experimental

A mixture of the isonicotinohydrazide (0.1 mol), and 5-methylfuran-2-carbaldehyde (0.1 mol) was stirred in refluxing ethanol (20 mL) for 4 h to afford the title compound (0.082 mol, yield 82%). Single crystals suitable for X-ray measurements were obtained by recrystallization from ethanol at room temperature.

### Refinement

All H atoms were fixed geometrically and allowed to ride on their attached atoms, with C—H distances in the range 0.93-0.97 Å and N—H = 0.86 Å, and with *U*_iso_=1.2–1.5*U*_eq_(N,C).

### Crystal data

**Table:**

C_12_H_11_N_3_O_2_	*Z* = 16
*M**_r_* = 229.24	*F*_000_ = 1920
Tetragonal, *I*4_1_/*a*	*D*_x_ = 1.290 Mg m^−^^3^
Hall symbol: -I 4ad	Mo *K*α radiation λ = 0.71073 Å
*a* = 17.313 (3) Å	Cell parameters from 4665 reflections
*b* = 17.313 (3) Å	θ = 2.9–27.2º
*c* = 15.749 (5) Å	µ = 0.09 mm^−^^1^
α = 90º	*T* = 273 (2) K
β = 90º	Block, yellow
γ = 90º	0.25 × 0.20 × 0.19 mm
*V* = 4720.5 (18) Å^3^	

### Data collection

**Table:**

Bruker SMART CCD area-detector diffractometer	2151 reflections with *I* > 2σ(*I*)
Radiation source: fine-focus sealed tube	*R*_int_ = 0.028
Monochromator: graphite	θ_max_ = 28.3º
*T* = 273(2) K	θ_min_ = 1.8º
φ and ω scans	*h* = −16→23
Absorption correction: none	*k* = −22→22
14901 measured reflections	*l* = −20→20
2911 independent reflections	

### Refinement

**Table:**

Refinement on *F*^2^	Hydrogen site location: inferred from neighbouring sites
Least-squares matrix: full	H-atom parameters constrained
*R*[*F*^2^ > 2σ(*F*^2^)] = 0.041	*w* = 1/[σ^2^(*F*_o_^2^) + (0.0456*P*)^2^ + 2.257*P*] where *P* = (*F*_o_^2^ + 2*F*_c_^2^)/3
*wR*(*F*^2^) = 0.117	(Δ/σ)_max_ < 0.001
*S* = 1.04	Δρ_max_ = 0.17 e Å^−^^3^
2911 reflections	Δρ_min_ = −0.14 e Å^−^^3^
155 parameters	Extinction correction: SHELXL97 (Sheldrick, 2008), Fc^*^=kFc[1+0.001xFc^2^λ^3^/sin(2θ)]^-1/4^
Primary atom site location: structure-invariant direct methods	Extinction coefficient: 0.0031 (3)
Secondary atom site location: difference Fourier map	

### Special details

**Table:**

Geometry. All e.s.d.'s (except the e.s.d. in the dihedral angle between two l.s. planes) are estimated using the full covariance matrix. The cell e.s.d.'s are taken into account individually in the estimation of e.s.d.'s in distances, angles and torsion angles; correlations between e.s.d.'s in cell parameters are only used when they are defined by crystal symmetry. An approximate (isotropic) treatment of cell e.s.d.'s is used for estimating e.s.d.'s involving l.s. planes.
Refinement. Refinement of *F*^2^ against ALL reflections. The weighted *R*-factor *wR* and goodness of fit *S* are based on *F*^2^, conventional *R*-factors *R* are based on *F*, with *F* set to zero for negative *F*^2^. The threshold expression of *F*^2^ > σ(*F*^2^) is used only for calculating *R*-factors(gt) *etc*. and is not relevant to the choice of reflections for refinement. *R*-factors based on *F*^2^ are statistically about twice as large as those based on *F*, and *R*- factors based on ALL data will be even larger.

### Fractional atomic coordinates and isotropic or equivalent isotropic displacement parameters (Å^2^)

**Table:**

	*x*	*y*	*z*	*U*_iso_*/*U*_eq_	
O2	0.58347 (5)	0.51303 (5)	0.10958 (6)	0.0482 (3)	
N3	0.72302 (6)	0.49350 (7)	0.03623 (7)	0.0427 (3)	
C12	0.67464 (8)	0.53721 (7)	−0.00223 (9)	0.0423 (3)	
H12A	0.6866	0.5578	−0.0552	0.051*	
N2	0.79076 (6)	0.47620 (7)	−0.00603 (7)	0.0443 (3)	
H2A	0.7980	0.4901	−0.0578	0.053*	
C8	0.60143 (8)	0.55421 (7)	0.03726 (8)	0.0417 (3)	
O1	0.83833 (7)	0.42110 (8)	0.11302 (7)	0.0750 (4)	
C4	0.91448 (8)	0.41092 (8)	−0.01179 (8)	0.0442 (3)	
C11	0.84487 (8)	0.43670 (9)	0.03721 (8)	0.0460 (3)	
C9	0.51105 (8)	0.53616 (9)	0.13450 (10)	0.0513 (4)	
C7	0.54259 (8)	0.60254 (8)	0.01805 (10)	0.0505 (4)	
H7A	0.5402	0.6366	−0.0276	0.061*	
C3	0.91374 (8)	0.39635 (10)	−0.09860 (9)	0.0531 (4)	
H3B	0.8696	0.4056	−0.1307	0.064*	
N1	1.04629 (8)	0.35518 (10)	−0.09513 (9)	0.0736 (5)	
C5	0.98256 (9)	0.39741 (11)	0.03194 (10)	0.0640 (5)	
H5A	0.9853	0.4061	0.0901	0.077*	
C2	0.98006 (9)	0.36780 (11)	−0.13609 (10)	0.0650 (5)	
H2B	0.9784	0.3566	−0.1938	0.078*	
C6	0.48512 (9)	0.59116 (9)	0.08124 (11)	0.0561 (4)	
H6A	0.4382	0.6170	0.0851	0.067*	
C1	1.04634 (10)	0.37078 (13)	−0.01203 (12)	0.0758 (6)	
H1B	1.0920	0.3633	0.0180	0.091*	
C10	0.47813 (11)	0.49172 (12)	0.20700 (12)	0.0760 (6)	
H10A	0.4277	0.5113	0.2203	0.114*	
H10B	0.5112	0.4971	0.2556	0.114*	
H10C	0.4743	0.4382	0.1918	0.114*	

### Atomic displacement parameters (Å^2^)

**Table:**

	*U*^11^	*U*^22^	*U*^33^	*U*^12^	*U*^13^	*U*^23^
O2	0.0471 (5)	0.0516 (6)	0.0459 (6)	0.0050 (4)	0.0087 (4)	0.0080 (4)
N3	0.0440 (6)	0.0466 (6)	0.0374 (6)	0.0030 (5)	0.0080 (5)	0.0042 (5)
C12	0.0482 (7)	0.0410 (7)	0.0377 (7)	−0.0013 (5)	0.0043 (5)	0.0035 (5)
N2	0.0455 (6)	0.0564 (7)	0.0309 (5)	0.0060 (5)	0.0089 (4)	0.0086 (5)
C8	0.0469 (7)	0.0390 (7)	0.0393 (7)	−0.0019 (5)	0.0027 (5)	0.0022 (5)
O1	0.0740 (8)	0.1156 (10)	0.0355 (6)	0.0391 (7)	0.0150 (5)	0.0225 (6)
C4	0.0432 (7)	0.0547 (8)	0.0346 (7)	0.0036 (6)	0.0027 (5)	0.0012 (6)
C11	0.0493 (8)	0.0564 (8)	0.0324 (7)	0.0078 (6)	0.0065 (5)	0.0053 (6)
C9	0.0462 (8)	0.0537 (8)	0.0540 (9)	0.0000 (6)	0.0107 (6)	−0.0028 (7)
C7	0.0529 (8)	0.0440 (7)	0.0545 (9)	0.0040 (6)	0.0006 (6)	0.0051 (6)
C3	0.0429 (7)	0.0785 (10)	0.0379 (7)	0.0050 (7)	−0.0012 (6)	−0.0047 (7)
N1	0.0497 (8)	0.1162 (13)	0.0550 (8)	0.0166 (8)	0.0024 (6)	−0.0196 (8)
C5	0.0577 (9)	0.0961 (13)	0.0381 (8)	0.0182 (9)	−0.0062 (7)	−0.0109 (8)
C2	0.0546 (9)	0.1006 (13)	0.0399 (8)	0.0090 (9)	0.0034 (7)	−0.0151 (8)
C6	0.0468 (8)	0.0544 (9)	0.0672 (10)	0.0085 (6)	0.0047 (7)	−0.0014 (7)
C1	0.0483 (9)	0.1212 (16)	0.0579 (10)	0.0220 (10)	−0.0099 (7)	−0.0188 (10)
C10	0.0718 (12)	0.0839 (13)	0.0722 (12)	0.0022 (9)	0.0292 (9)	0.0134 (10)

### Geometric parameters (Å, °)

**Table:**

O2—C9	1.3735 (16)	C7—C6	1.421 (2)
O2—C8	1.3793 (16)	C7—H7A	0.9300
N3—C12	1.2812 (17)	C3—C2	1.383 (2)
N3—N2	1.3813 (15)	C3—H3B	0.9300
C12—C8	1.4421 (18)	N1—C2	1.334 (2)
C12—H12A	0.9300	N1—C1	1.336 (2)
N2—C11	1.3450 (17)	C5—C1	1.383 (2)
N2—H2A	0.8600	C5—H5A	0.9300
C8—C7	1.3526 (19)	C2—H2B	0.9300
O1—C11	1.2293 (16)	C6—H6A	0.9300
C4—C5	1.385 (2)	C1—H1B	0.9300
C4—C3	1.3904 (19)	C10—H10A	0.9600
C4—C11	1.4990 (18)	C10—H10B	0.9600
C9—C6	1.346 (2)	C10—H10C	0.9600
C9—C10	1.490 (2)		
			
C9—O2—C8	106.92 (11)	C2—C3—C4	118.50 (14)
C12—N3—N2	117.08 (11)	C2—C3—H3B	120.7
N3—C12—C8	119.42 (12)	C4—C3—H3B	120.7
N3—C12—H12A	120.3	C2—N1—C1	116.18 (14)
C8—C12—H12A	120.3	C1—C5—C4	119.14 (14)
C11—N2—N3	117.23 (11)	C1—C5—H5A	120.4
C11—N2—H2A	121.4	C4—C5—H5A	120.4
N3—N2—H2A	121.4	N1—C2—C3	124.47 (14)
C7—C8—O2	109.55 (12)	N1—C2—H2B	117.8
C7—C8—C12	133.77 (13)	C3—C2—H2B	117.8
O2—C8—C12	116.66 (11)	C9—C6—C7	107.52 (13)
C5—C4—C3	117.78 (13)	C9—C6—H6A	126.2
C5—C4—C11	118.59 (12)	C7—C6—H6A	126.2
C3—C4—C11	123.56 (12)	N1—C1—C5	123.88 (15)
O1—C11—N2	122.64 (12)	N1—C1—H1B	118.1
O1—C11—C4	120.56 (13)	C5—C1—H1B	118.1
N2—C11—C4	116.79 (11)	C9—C10—H10A	109.5
C6—C9—O2	109.43 (13)	C9—C10—H10B	109.5
C6—C9—C10	135.66 (15)	H10A—C10—H10B	109.5
O2—C9—C10	114.68 (14)	C9—C10—H10C	109.5
C8—C7—C6	106.55 (13)	H10A—C10—H10C	109.5
C8—C7—H7A	126.7	H10B—C10—H10C	109.5
C6—C7—H7A	126.7		

### Hydrogen-bond geometry (Å, °)

**Table:**

*D*—H···*A*	*D*—H	H···*A*	*D*···*A*	*D*—H···*A*
N2—H2A···O1^i^	0.86	2.18	2.9083 (19)	143
N2—H2A···N3^i^	0.86	2.58	3.3255 (19)	146

Symmetry codes: (i) −*y*+5/4, *x*−1/4, *z*−1/4.
